# Effects of mindfulness yoga intervention on physical function, anxiety, inflammatory response, and relapse intention in methamphetamine addicts: a 24-week randomized controlled trial

**DOI:** 10.3389/fpubh.2026.1788887

**Published:** 2026-04-14

**Authors:** Su Xia Chen, Zhi Xue Lyu, Zong Ji Hao, Qiang Tang, Wan Jun Li

**Affiliations:** 1 School of Educational Science, Xinjiang Normal University, Xinjiang, Urumqi, China; 2 College of Marxism, Xinjiang Medical University, Ürümqi, China; 3 Biology Teaching and Research Group, Chongqing Tongnan Experimental High School, Chongqing, China; 4 School of Physical Education, Southwest University, Chongqing, China

**Keywords:** anxiety, inflammatory response, methamphetamine addicts, mindful yoga, neurotransmitters, physical function, relapse intention

## Abstract

**Background:**

Methamphetamine (MA) addiction is a chronic relapsing disorder that impairs physical health, induces mental distress (e.g., anxiety), and triggers abnormal inflammatory and neurotransmitter responses, all contributing to high relapse rates. Mindfulness and yoga interventions may alleviate psychological distress and improve physical well-being in substance users; however, few studies have systematically investigated their comprehensive effects on physical function, inflammatory markers, neurotransmitter balance, and relapse intention in MA addicts, leaving a gap in non-pharmacological interventions for this population.

**Objective:**

This randomized controlled trial aimed to investigate the effects of a 24-week yoga intervention integrated with mindfulness components on multiple health outcomes among individuals with methamphetamine use disorder, ncluding physical function (assessed by Functional Movement Screening [FMS], reaction time, sleep quality, subjective fatigue, and creatine kinase [CK]), anxiety, inflammatory response (tumor necrosis factor-*α* [TNF-α], interleukin-6 [IL-6], and interleukin-10 [IL-10]), heart rate variability (HRV), relapse-related neurotransmitters (dopamine [DA], serotonin [5-HT], and norepinephrine [NE]), and relapse intention.

**Methods:**

Eighty MA addicts meeting DSM-5 criteria were randomly assigned to an experimental group (*n* = 41; 40-min mindfulness yoga sessions, three times weekly for 24 weeks) or a control group (*n* = 39; no additional intervention). Assessments were conducted at baseline and post-intervention for all outcomes, with additional measurements at 4 and 12 weeks for fatigue, CK, and inflammatory markers.

**Results:**

Following the 24-week intervention, the experimental group demonstrated significant improvements in sleep quality (PSQI score reduced from 14.32 to 8.41), anxiety levels (BAI score decreased), FMS total score (increased from 9.24 to 12.12), and reaction time (improved from 0.59 s to 0.48 s) compared to baseline and controls (*p* < 0.01). Relapse intention (OCDS score reduced from 29.12 to 18.45) and NE levels significantly decreased, while DA and 5-HT levels significantly increased (*p* < 0.01). The experimental group also showed enhanced parasympathetic activity (higher HF index, lower LF/HF ratio; both *p* < 0.01), reduced CK, TNF-*α*, IL-6, and subjective fatigue, and elevated IL-10 levels (*p* < 0.05).

**Conclusion:**

Compared with a traditional relaxation and stretching control condition, mindfulness yoga intervention demonstrated superior efficacy in improving physical function, sleep quality, and reaction ability of MA addicts. These findings support mindfulness yoga as a promising non-pharmacological intervention for MA addiction rehabilitation.

**Clinical trial registration:**

https://www.chictr.org.cn/showproj.html?proj=40393, Unique Identifier is ChiCTR1900024439.

## Introduction

Methamphetamine (MA) addiction has become a serious public health problem worldwide due to its strong addiction, high relapse rate, and severe damage to physical and mental health (1). Conceptually, MA addiction is a complex neuropsychiatric disorder characterized by compulsive drug-seeking behavior, loss of control over use, and persistent craving—rooted in neurobiological adaptations and psychological vulnerabilities (2). Neurobiologically, long-term MA exposure induces structural and functional alterations in brain regions involved in reward processing (ventral tegmental area, nucleus accumbens), executive control (prefrontal cortex, PFC), and stress regulation (amygdala, hypothalamic–pituitary–adrenal axis) (3). Psychologically, MA addicts often exhibit comorbid mental health issues (anxiety, depression), impaired self-regulation, and heightened impulsivity—all of which form a vicious cycle reinforcing addiction and relapse (4).

Long-term MA use leads to systemic damage: on the physical level, it causes skeletal muscle microdamage, chronic inflammation, decreased motor function, and sleep disorders (5); on the psychological level, it induces anxiety, depression, and intense drug cravings, which are key factors leading to relapse (6); on the neurochemical level, it disrupts the balance of neurotransmitters such as dopamine (DA), serotonin (5-HT), and norepinephrine (NE) in the brain—DA depletion reduces reward sensitivity and reinforces drug-seeking to compensate for blunted natural rewards (7), 5-HT deficiency exacerbates emotional disorders and impairs impulse control (4), and NE overactivation enhances stress response and craving reactivity (8). These neurochemical imbalances are closely intertwined with behavioral manifestations of addiction: cravings (a state of motivational urgency to use drugs) and impulsivity (a tendency to act without foresight, linked to PFC hypofunction) (9, 10). Both constructs are core components of addiction maintenance and relapse, as they reflect dysregulated neurocognitive processes mediated by PFC-related neural networks (9). DA depletion reduces reward sensitivity, 5-HT deficiency exacerbates emotional disorders, and NE overactivation enhances stress response, all of which jointly increase relapse risk (11).

Existing rehabilitation interventions for MA addicts mainly include pharmacotherapy and cognitive-behavioral therapy, but they have limitations such as side effects, high cost, and difficulty in maintaining long-term effects (12). In recent years, complementary and alternative therapies have attracted attention. Yoga, as a mind–body practice integrating physical postures (asana), breath control (pranayama), and meditative awareness, exerts multifaceted effects through physical, neuroimmunological, and neurobiological mechanisms (13). Physically, yoga improves mitochondrial function and reduces chronic inflammation—addressing MA-induced skeletal muscle damage (14); neurobiologically, it modulates neurotransmitter systems (e.g., increasing DA and 5-HT levels) and enhances PFC activity—improving executive control (15); psychologically, it fosters emotional regulation and mindfulness—alleviating anxiety/depression (16). Mindfulness training, meanwhile, strengthens metacognitive awareness and reduces automaticity of craving responses—enhancing self-control (6).

Studies have shown that yoga can improve mental health by regulating neurotransmitters and reducing stress (16), and mindfulness training can enhance self-control and reduce addictive cravings (6). Cumulative evidence supports the efficacy of yoga and mindfulness (separately) in addiction intervention: qualitative research (17) found that yoga promotes “life transformation” among substance users by fostering self-compassion and breaking habitual thought patterns; a pilot study on Raja yoga meditation (18) reported reduced relapse rates among MA addicts when combined with medication-assisted treatment (19); observed significant reductions in cravings, impulsivity, and psychiatric symptoms in women with substance use disorders (SUDs) receiving adjunctive yoga during inpatient treatment. For mindfulness-based interventions (20), demonstrated that mindfulness-based relapse prevention (MBRP) reduced relapse intention and improved emotional stability in Chinese MA-dependent patients, while (21) reported enhanced distress tolerance and reduced temptation in MA addicts following MBRP. However, few studies have integrated these two approaches into mindfulness yoga, nor systematically explored its neurochemical mechanisms (e.g., regulation of DA/5-HT/NE) in MA addiction—creating a critical research gap.

However, few studies have explored the effects of mindfulness yoga (integrating mindfulness and yoga) on MA addicts, especially its regulatory effects on relapse intention and related neurotransmitters. To fill this gap, this study designed a randomized controlled experiment to explore the value of mindfulness yoga in improving the physical and mental health and reducing relapse risk of MA addicts.

Inclusion and exclusion criteria were refined to control for physical activity confounders: Participants were required to be sedentary (defined as minutes of moderate-intensity exercise per week) for at least 3 months prior to enrollment, with no history of structured exercise (e.g., gym training, sports clubs) or yoga/mindfulness practice in the past year. Concurrent participation in other physical activity programs during the study period was prohibited, and fitness level was assessed using the 6-min walk test to ensure homogeneity across groups. This control addresses potential confounding from non-intervention physical activity, strengthening causal inference regarding mindfulness yoga’s effects (22).

Outcome measures were selected based on theoretical and empirical rationales: (1) Neurotransmitters (DA, 5-HT, NE): Selected due to their well-documented role in MA-induced addiction pathophysiology—DA for reward dysregulation, 5-HT for emotional/impulsive control, and NE for stress-craving pathways (4, 7, 8, 11); (2) Relapse intention: Measured via the Relapse Risk Questionnaire (7), as it directly reflects motivational vulnerability to relapse; (3) Cravings: Assessed using the Methamphetamine Craving Questionnaire (23), given its status as a proximal predictor of relapse (6); (4) Impulsivity: Evaluated via the Barratt Impulsiveness Scale (24), as it mediates the relationship between neurochemical imbalance and compulsive drug-seeking; (5) Physical health indicators (muscle damage markers, inflammatory cytokines): Included to address MA-induced systemic impairment. Psychometric properties of all measures were verified in prior SUD research, ensuring reliability and validity.

## Materials and methodology

### Method section

#### Study population

**Figure 1:**
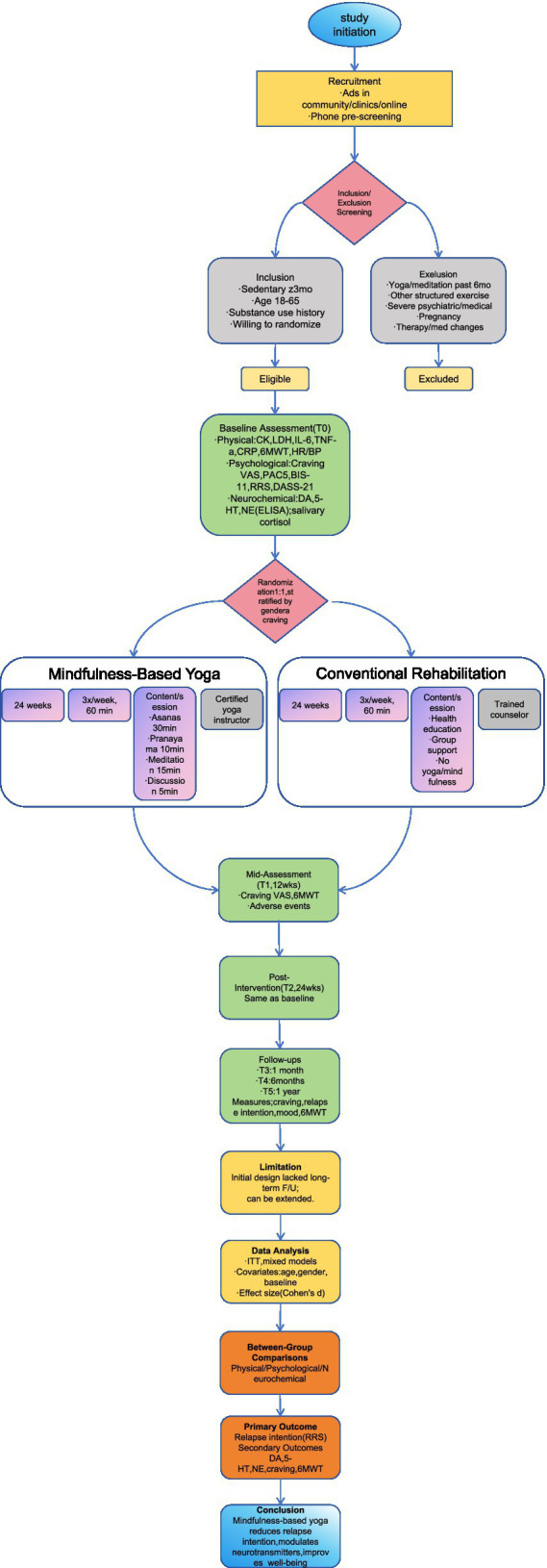
Study flow diagram of the randomized controlled trial.

This study was registered in the Chinese Clinical Trial Registry (ChiCTR1900024439) and conducted at a compulsory isolation drug rehabilitation center in Chongqing. Participants were recruited from March to April 2022, the intervention was implemented from April to October 2022, and all participants underwent a rigorous multi-stage screening process. Eligible participants provided written informed consent and were assessed using the Mini-Mental State Examination (MMSE) (score ≥24) to ensure they had sufficient cognitive ability to understand the study content. The flowchart (Figure 1) illustrates the entire study process.

#### Sample size and attrition

Based on calculations using GPower 3.1 software, a minimum sample size of 36 was required for a repeated measures analysis of variance (RM-ANOVA) with *α* = 0.05, medium effect size (*f* = 0.25), and 0.95 statistical power. Considering a 10% attrition rate, 40 participants per group were targeted. Initially, 84 MA addicts were enrolled, and 4 withdrew due to severe medical complications (2 with acute pancreatitis, 1 with severe arrhythmia, and 1 with acute exacerbation of schizophrenia, all diagnosed by tertiary hospitals). The final sample included 80 participants (41 in the experimental group and 39 in the control group). Baseline data of withdrawers showed no significant differences from completers (*p* > 0.05), indicating no impact on group balance.

### Inclusion and exclusion criteria

#### Inclusion criteria

Meet DSM-5 diagnostic criteria for methamphetamine use disorder, with a withdrawal period ≥2 weeks (to exclude acute withdrawal reactions);

Aged 18–45 years;

No severe mental disorders, neurological diseases, or severe organ dysfunction (confirmed by medical examinations);

No history of mindfulness, yoga, or meditation training in the past year (confirmed by questionnaire);

No engagement in structured exercise or sports within 3 months prior to enrollment or during the study period (confirmed by questionnaire);

Voluntarily participate and sign informed consent (guardian consent for participants under 20 years old), with MMSE score ≥ 24.

#### Exclusion criteria

Fail to meet DSM-5 diagnostic criteria for methamphetamine use disorder or withdrawal period < 2 weeks;

Aged < 18 or > 45 years;

Presence of severe mental, neurological, or organ dysfunction;

History of mindfulness, yoga, or meditation training in the past year;

Engagement in structured exercise or sports within 3 months prior to enrollment or during the study period;

Refuse to sign informed consent or inability to complete assessments due to cognitive impairment (MMSE score < 24).

### Group randomization and balance check

Stratified randomization was used, with age (18–30 years, 31–45 years) and addiction duration (<5 years, ≥5 years) as stratification factors. A statistician independent of recruitment and assessment generated a random allocation sequence using the stratified random sampling module in SPSS 22.0. Group assignment was concealed using sealed envelopes, which were opened by the intervention implementer before the start of the intervention. Baseline comparisons of demographic data (age, addiction duration) and all outcome indicators (FMS score, reaction time, Pittsburgh Sleep Quality Index [PSQI] score, Beck Anxiety Inventory [BAI] score, Obsessive-Compulsive Drug Craving Scale [OCDS] score, neurotransmitters, inflammatory factors, heart rate variability [HRV]) between the two groups showed no significant differences (all *p* > 0.05), confirming the effectiveness of randomization and ensuring no baseline confounding between groups.

### Intervention measures

Yoga is a mind–body practice that integrates physical postures, breathing techniques, and mindful awareness, with different styles varying in emphasis on movement intensity, meditative components, and therapeutic goals. Unlike more dynamic styles such as Vinyasa yoga (focused on flowing movements) or Pranayama (focused solely on breath control), the mindfulness yoga used in this study is a specialized mind–body intervention that combines gentle physical postures with breath-focused meditation and non-judgmental awareness of thoughts and bodily sensations—distinctive for its emphasis on integrating mindfulness into every aspect of the practice, rather than prioritizing movement intensity or breath manipulation alone.

The underlying neural and physiological mechanisms of this mindfulness yoga intervention include regulation of PFC-related neural networks (which subserve cravings and impulsivity), modulation of neurotransmitter levels (e.g., dopamine, serotonin) associated with reward and mood, reduction of inflammatory responses, and improvement of autonomic nervous system balance. These mechanisms are particularly relevant to MA addiction, as they target the core neurobiological and psychological impairments of the disorder, including dysregulated craving, increased impulsivity, and neuroinflammation.

### Experimental group: mindfulness yoga intervention

The intervention was conducted by two yoga instructors certified by the International Yoga Alliance (RYT 500-h) and trained in Level 2 Mindfulness-Based Cognitive Therapy (MBCT), both with over 3 years of experience working with addiction populations. Prior to the study, the instructors underwent standardized training to ensure consistency in movement guidance, verbal cues, and mindfulness instructions, with an inter-rater reliability of Kappa = 0.89. The intervention was carried out in a dedicated yoga studio with a controlled environment (temperature: 22–25 °C, humidity: 50–60%, ambient noise ≤ 40 dB), equipped with yoga mats, bolsters, and blocks to support participants with varying flexibility.

The 40-min mindfulness yoga session (conducted 3 times per week for 24 weeks, totaling 72 sessions) consisted of five phases: preparation phase (5 min) for breathing regulation and session orientation; body scan (10 min) for supine awareness of bodily sensations; sitting meditation (10 min) for breath-focused meditation with acceptance of distracting thoughts and drug cravings; gentle yoga asanas (15 min) including supine stretch, cat-cow pose, child’s pose, seated forward fold, and easy twist, performed with synchronized breathing to avoid breath-holding or overstretching; and mindful walking (5 min) for slow walking with attention to foot-ground contact.

Previous research has shown that similar mindfulness-based yoga interventions can effectively reduce cravings and impulsivity in individuals with substance use disorders, supporting the selection of this specific practice. Adherence was monitored via sign-in, with one-on-one supplementary intervention provided within 48 h for participants who missed sessions due to valid reasons (e.g., mild illness). Participants who missed more than 6 sessions (attrition rate >8.3%) were excluded. The average adherence rate in the experimental group was 92.5%.

### Control group: traditional relaxation training

The intervention was delivered by the same instructors as the experimental group (without mindfulness training), with consistent intervention environment, duration, and frequency to ensure equivalence of non-intervention factors. The 40-min session (conducted 3 times per week for 24 weeks) included three phases: preparation phase (5 min) identical to the experimental group; muscle stretching (20 min) involving gentle stretching of the neck, shoulders, back, legs, and waist, synchronized with breathing; and deep breathing training (15 min) including diaphragmatic, thoracic, and combined breathing, focusing on breathing depth without mindfulness-based awareness or thought acceptance.

Adherence monitoring was consistent with the experimental group, with an average adherence rate of 91.8%.

### Unified management of confounding factors

This unified management also ensured that no participants engaged in additional structured exercise or mindfulness practices outside of the assigned intervention, further controlling for potential confounders. During the 24-week intervention, all participants received unified management of daily activities (e.g., scheduled work, rest, and recreational time), diet (standardized three meals a day, low-fat, high-fiber, no caffeine or alcohol), and medication (no psychotropic drugs or drugs affecting neurotransmitters or inflammatory factors) to minimize confounding effects.

### Test methods

Outcome indicators were selected based on theoretical and empirical evidence linking mindfulness yoga to methamphetamine (MA) addiction core impairments: cravings and impulsivity (core indicators, associated with prefrontal cortex networks and relapse); neurotransmitters (DA, 5-HT, NE, involved in reward, craving and emotion); inflammatory factors (TNF-*α*, IL-6, IL-10, for neuroinflammation); and FMS, reaction time, fatigue, sleep quality, anxiety, HRV (for physical, cognitive, emotional and autonomic function, all impaired in MA-dependent individuals and potentially improved by mindfulness yoga).

### Blood index detection

Venous blood (5 mL) was collected at baseline, 4, 12 and 24 weeks (6:30–7:30 a.m., 12-h fast). After 30-min room temperature standing, samples were centrifuged (3,000 r/min, 10 cm radius, 10 min) to separate serum, aliquoted into EP tubes (200 μL/tube), stored at −80 °C (≤1 freeze–thaw cycle), labeled for traceability, and transported with dry ice.

CK, TNF-*α*, IL-6, IL-10 (R&D Systems) and DA, 5-HT, NE (Abcam) were detected via ELISA (strictly per instructions). Quality controls were included; detection was performed with Thermo Scientific Multiskan FC (corresponding wavelengths). Intra-assay CV < 5% and inter-assay CV < 8% ensured accuracy.

### Functional movement screening (FMS)

Two FMS-certified, group-blinded assessors evaluated MA-dependent individuals’ basic physical function using 7 standardized movement patterns (1–3 points). Pre-study training was provided; tests were video-recorded. Discrepancies ≥1 point were resolved via video review. Inter-rater reliability ICC = 0.91.

### Fatigue assessment

Psychological fatigue was assessed with the validated Chinese Athlete Psychological Fatigue Scale (Cronbach’s *α* = 0.86; 15 items, 1–5 Likert scale; higher scores = more severe fatigue). Questionnaires were completed quietly with 100% recovery.

### Reaction time test

Cognitive impulsivity was assessed with FYS-I electronic reaction time tester (≤1 ms response, ±0.001 s error). Participants (50 cm from screen) completed 5 practices and 10 formal trials; average was final result (invalid trials repeated).

### Sleep quality assessment

Sleep quality was assessed with Chinese PSQI (Cronbach’s *α* = 0.82; 18 items, 7 dimensions, 0–21 total score; higher scores = poorer sleep) 1 week pre- and post-intervention (past month sleep), assisted by trained researchers.

### Anxiety assessment

Anxiety was assessed with Chinese BAI (Cronbach’s *α* = 0.85; 21 items, 0–3 Likert scale; higher scores = more severe anxiety). Participants completed quietly based on current feelings (≈8 min).

### Relapse intention assessment

Relapse intention was assessed with Chinese OCDS (Cronbach’s α = 0.88; 14 items, 0–4 Likert scale; higher scores = stronger intention), completed simultaneously with BAI without external interference.

### Heart rate variability (HRV) test

Autonomic balance was assessed with Maidic SA-3000P HRV analyzer (1,000 Hz sampling; time-domain: SDNN, RMSSD; frequency-domain: LF 0.04–0.15 Hz, HF 0.15–0.4 Hz, LF/HF). Participants rested 10 min (quiet, seated, eyes closed), wore chest monitor; 4-min data were collected (abnormal beats excluded, ≥3 min valid retained).

## Data analysis

All statistical analyses were conducted by a statistician blinded to group assignments to reduce bias. SPSS 22.0 was used for statistical analysis, and results were expressed as M ± SD (95% CI). Normality and homogeneity of variance tests were performed prior to formal analysis. Independent sample t-tests were used for inter-group comparisons, paired sample t-tests for intra-group comparisons, and general linear ANOVA for multi-time point data (to assess changes in outcomes over the 24-week intervention period). Significance levels were set as *p* < 0.01 (very significant) and 0.01 < *p* < 0.05 (significant). Significance levels were set as *p* < 0.01 (very significant) and 0.01 < *p* < 0.05 (significant). Given the exploratory nature of the study and the range of outcome measures, no correction for multiple comparisons (e.g., Bonferroni) was applied to balance the risk of Type I and Type II errors; thus, the findings should be interpreted with caution.

## Results

### Changes in relapse intention before and after the experiment

At baseline, no significant difference in relapse intention was observed between the two groups (*p* > 0.05). After 24 weeks, the experimental group showed a significant reduction in OCDS scores compared with both its own baseline and the control group (both *p* < 0.01), with a large effect size (Cohen’s *d* = 0.85). No significant change was observed in the control group (*p* > 0.05) (Table 1).

**Table 1:** Comparison of relapse intention before and after the experiment.

Variable	Group	Before the experiment	Post-experiment	*T*	*p*	Effect size
Relapse intention (OCDS score)	Control group	28.67 ± 4.37 (27.35, 30.00)	27.89 ± 4.12 (26.61, 29.17)	1.02	0.31	0.11
	Experimental group	29.12 ± 4.01 (27.98, 30.26)	18.45 ± 3.26 (17.38, 19.52)	15.68	0.00	0.85

### Changes in relapse-related neurotransmitters before and after the experiment

Baseline levels of DA, 5-HT, and NE were comparable between groups (all *p* > 0.05). After 24 weeks, the experimental group showed a significant increase in DA and 5-HT levels and a significant decrease in NE levels compared with both baseline and the control group (all *p* < 0.01). Effect sizes were large (*d* = 0.82, 0.88, and 0.92, respectively). No significant changes were observed in the control group (all *p* > 0.05) (Table 2).

**Table 2:** Comparison of relapse-related neurotransmitters before and after the experiment.

Variable	Group	Before the experiment	Post-experiment	*T*	*p*	Effect size
DA (ng/mL)	Control group	12.35 ± 2.17 (11.68, 13.02)	13.12 ± 2.34 (12.41, 13.83)	1.65	0.10	0.18
	Experimental group	12.18 ± 2.05 (11.55, 12.81)	18.67 ± 2.58 (17.82, 19.52)	14.29	0.00	0.82
5-HT (ng/mL)	Control group	35.62 ± 4.87 (34.21, 37.03)	37.15 ± 5.02 (35.68, 38.62)	1.53	0.13	0.16
	Experimental group	34.98 ± 4.65 (33.65, 36.31)	48.32 ± 5.36 (46.75, 49.89)	16.83	0.00	0.88
NE (ng/mL)	Control group	89.45 ± 8.67 (86.89, 92.01)	87.62 ± 8.34 (85.15, 90.09)	1.12	0.27	0.12
	Experimental group	90.12 ± 8.95 (87.48, 92.76)	65.38 ± 7.52 (63.21, 67.55)	18.56	0.00	0.92

### Changes in sleep quality of subjects before and after the experiment

Baseline PSQI scores did not differ significantly between groups (*p* > 0.05). After 24 weeks, the experimental group showed a significant reduction in PSQI scores compared with baseline and the control group (both *p* < 0.01), with a large effect size (*d* = 0.78). No significant change was observed in the control group (*p* > 0.05) (Table 3).

**Table 3:** Comparison of sleep quality before and after the experiment.

Variable name	Group	Before the experiment	Post-experiment	*T*	*p*	Effect size
Sleep quality (PSQI score)	Control group	14.05 ± 3.01 (13.12, 14.98)	13.68 ± 2.87 (12.81, 14.55)	0.65	0.52	0.07
	Experimental group	14.32 ± 2.95 (13.45, 15.19)	8.41 ± 2.52 (7.76, 9.06)	12.38	0.00	0.78

Compared with the control group, represents *p* < 0.01; compared with before the experiment, represents *p* < 0.01.

### Changes in the reaction time of the subjects before and after the experiment

Baseline reaction time was comparable between groups (*p* > 0.05). After 24 weeks, the experimental group showed a significant reduction in reaction time compared with baseline and the control group (both *p* < 0.01), with a moderate-to-large effect size (*d* = 0.65). The control group showed a marginal improvement (*p* = 0.02, *d* = 0.25) (Table 4).

**Table 4:** Comparison of reaction time of subjects before and after the experiment.

Variable name	Group	Before the experiment	Post-experiment	*T*	*p*	Effect size
Reaction time (s)	Control group	0.58 ± 0.15 (0.54, 0.62)	0.55 ± 0.13 (0.51, 0.59)	2.41	0.02	0.25
	Experimental group	0.59 ± 0.16 (0.55, 0.63)	0.48 ± 0.11 (0.45, 0.51)	6.89	0.00	0.65

### Changes in FMS test scores of subjects before and after the experiment

Baseline FMS scores for all seven components and total scores were comparable between groups (all *p* > 0.05). After 24 weeks, the experimental group showed significant improvements in all FMS components and total scores compared with baseline and the control group (all *p* < 0.01), with effect sizes ranging from *d* = 0.47 to 0.75. No significant changes were observed in the control group (all *p* > 0.05) (Table 5).

**Table 5:** Comparison of FMS scores of subjects before and after the experiment.

Group	Squat		Hurdle step		Lunge		Shoulder mobility		Active straight-leg raise		Trunk stability push-up		Rotary stability		Total score	
	Before	Post-experiment	Before	Post-experiment	Before	Post-experiment	Before	Post-experiment	Before	Post-experiment	Before	Post-experiment	Before	Post-experiment	Before	Post-experiment
Control group	1.42 ± 0.38 (1.31, 1.53)	1.38 ± 0.41 (1.26, 1.50)	1.37 ± 0.45 (1.25, 1.49)	1.32 ± 0.43 (1.20, 1.44)	1.28 ± 0.42 (1.17, 1.39)	1.25 ± 0.40 (1.14, 1.36)	1.35 ± 0.44 (1.23, 1.47)	1.30 ± 0.42 (1.18, 1.42)	1.25 ± 0.40 (1.14, 1.36)	1.22 ± 0.38 (1.11, 1.33)	1.30 ± 0.41 (1.19, 1.41)	1.27 ± 0.39 (1.16, 1.38)	1.22 ± 0.39 (1.11, 1.33)	1.19 ± 0.37 (1.08, 1.30)	9.39 ± 1.87 (8.85, 9.93)	9.13 ± 1.76 (8.62, 9.64)
Experimental group	1.40 ± 0.39 (1.29, 1.51)	1.82 ± 0.46 (1.69, 1.95)	1.35 ± 0.43 (1.23, 1.47)	1.79 ± 0.48 (1.66, 1.92)	1.26 ± 0.41 (1.15, 1.37)	1.75 ± 0.45 (1.63, 1.87)	1.32 ± 0.42 (1.20, 1.44)	1.71 ± 0.44 (1.59, 1.83)	1.23 ± 0.38 (1.12, 1.34)	1.68 ± 0.43 (1.56, 1.80)	1.28 ± 0.39 (1.17, 1.39)	1.65 ± 0.42 (1.53, 1.77)	1.20 ± 0.37 (1.09, 1.31)	1.62 ± 0.40 (1.51, 1.73)	9.24 ± 1.78 (8.73, 9.75)	12.12 ± 1.95 (11.53, 12.71)
*t*	0.28	−5.82	0.24	−6.13	0.25	−6.57	0.31	−5.98	0.26	−5.67	0.23	−5.42	0.27	−4.98	0.35	−7.89
*p*	0.78	0.00	0.81	0.00	0.80	0.00	0.76	0.00	0.79	0.00	0.82	0.00	0.79	0.00	0.73	0.00
Effect size	0.03	0.56	0.02	0.59	0.02	0.63	0.03	0.57	0.02	0.54	0.02	0.51	0.03	0.47	0.04	0.75

### Changes in fatigue level of subjects at different stages

At baseline, subjective fatigue scores and CK levels were comparable between groups (both *p* > 0.05). Across the 24-week intervention, the experimental group showed significantly lower subjective fatigue scores and CK levels at 4, 12, and 24 weeks compared with the control group (all *p* < 0.01). A pattern of initial increase followed by progressive decline was observed in both groups, with the experimental group demonstrating sustained improvement over time (Table 6).

**Table 6:** Comparison of fatigue level and CK at different stages of the experiment.

Variable	Group	Pre	4 weeks	12 weeks	24 weeks
Subjective fatigue score	Control group	38.67 ± 5.37 (37.12, 40.22)	42.35 ± 5.62 (40.71, 43.99)	40.12 ± 5.48 (38.55, 41.69)	37.89 ± 5.26 (36.37, 39.41)
	Experimental group	39.12 ± 5.15 (37.65, 40.59)	35.48 ± 4.98 (34.08, 36.88)	31.25 ± 4.65 (29.95, 32.55)	27.68 ± 4.32 (26.52, 28.84)
*t*/*p* (24 weeks)		0.45/0.65	5.87/0.00	7.92/0.00	9.65/0.00
CK (U/L)	Control group	385.62 ± 65.87 (366.21, 405.03)	452.35 ± 72.48 (430.15, 474.55)	421.68 ± 68.95 (401.52, 441.84)	398.75 ± 66.32 (379.21, 418.29)
	Experimental group	392.15 ± 68.45 (372.08, 412.22)	378.42 ± 63.25 (360.58, 396.26)	325.78 ± 59.68 (309.42, 342.14)	289.36 ± 56.42 (275.28, 303.44)
*t*/*p* (24 weeks)		0.49/0.62	5.12/0.00	7.35/0.00	8.89/0.00

### Changes in inflammation levels of subjects at different stages

Baseline levels of TNF-*α*, IL-6, and IL-10 were comparable between groups (all *p* > 0.05). After 24 weeks, the experimental group showed significant reductions in TNF-α and IL-6 levels and a significant increase in IL-10 levels compared with the control group (all *p* < 0.01). These differences were already evident at 4 weeks and progressively increased over the intervention period (Table 7).

**Table 7:** Comparison of inflammation levels at different stages of the experiment.

Variable	Group	Pre	4 weeks	12 weeks	24 weeks
TNF-α (pg/mL)	Control group	28.65 ± 4.87 (27.21, 30.09)	35.28 ± 5.62 (33.51, 37.05)	32.15 ± 5.38 (30.48, 33.82)	29.87 ± 4.95 (28.45, 31.29)
	Experimental group	29.12 ± 5.02 (27.63, 30.61)	27.45 ± 4.78 (26.08, 28.82)	23.68 ± 4.35 (22.45, 24.91)	20.35 ± 3.98 (19.32, 21.38)
*t*/*p* (24 weeks)		0.46/0.65	6.23/0.00	7.89/0.00	9.76/0.00
IL-6 (pg/mL)	Control group	18.35 ± 3.62 (17.32, 19.38)	24.12 ± 4.25 (12.87, 25.37)	21.68 ± 3.98 (20.65, 22.71)	19.87 ± 3.75 (18.85, 20.89)
	Experimental group	18.72 ± 3.78 (17.65, 19.79)	16.85 ± 3.42 (15.92, 17.78)	13.42 ± 3.15 (12.65, 14.19)	10.25 ± 2.89 (9.62, 10.88)
*t*/*p* (24 weeks)		0.48/0.63	7.15/0.00	8.62/0.00	10.35/0.00
IL-10 (pg/mL)	Control group	8.35 ± 1.62 (7.82, 8.88)	7.12 ± 1.45 (6.65, 7.59)	7.68 ± 1.58 (7.18, 8.18)	8.15 ± 1.60 (7.63, 8.67)
	Experimental group	8.12 ± 1.58 (7.61, 8.63)	9.45 ± 1.72 (8.92, 9.98)	11.68 ± 1.85 (11.08, 12.28)	13.35 ± 2.01 (12.68, 14.02)
*t*/*p* (24 weeks)		0.51/0.61	5.98/0.00	8.25/0.00	9.89/0.00

### Changes in heart rate variability of subjects before and after the experiment

Baseline HRV parameters were comparable between groups (all *p* > 0.05). After 24 weeks, the experimental group showed a significant increase in HF power and a significant decrease in LF/HF ratio compared with the control group (both *p* < 0.01), with large effect sizes (*d* = 0.57 and 0.79, respectively). No significant changes were observed in SDNN, RMSSD, or LF between groups (all *p* > 0.05) (Table 8).

**Table 8:** Changes of HRV index of subjects in two groups before and after intervention.

Group	SDNN		RMSSD		LF		HF		LF/HF	
	Before	Post-experiment	Before	Post-experiment	Before	Post-experiment	Before	Post-experiment	Before	Post-experiment
Control group	42.09 ± 12.45 (38.21, 45.97)	45.68 ± 14.70 (41.05, 50.31)	40.62 ± 10.44 (37.52, 43.72)	41.35 ± 11.51 (38.08, 44.62)	185.32 ± 52.46 (170.15, 200.49)	192.65 ± 58.72 (176.82, 208.48)	210.45 ± 67.68 (188.21, 232.69)	225.36 ± 72.81 (201.53, 249.19)	0.89 ± 0.27 (0.81, 0.97)	0.85 ± 0.25 (0.77, 0.93)
Experimental group	41.25 ± 11.85 (37.58, 44.92)	47.34 ± 13.28 (43.12, 51.56)	39.87 ± 10.62 (36.75, 42.99)	45.68 ± 11.22 (42.55, 48.81)	180.73 ± 50.72 (165.98, 195.48)	175.46 ± 48.65 (161.82, 189.10)	205.90 ± 65.88 (184.35, 227.45)	312.64 ± 78.43 (288.52, 336.76)	0.92 ± 0.29 (0.84, 1.00)	0.56 ± 0.18 (0.50, 0.62)
*t*	0.32	0.58	0.35	1.82	0.45	1.56	0.38	5.98	0.51	8.76
*p*	0.32/0.75	0.58/0.56	0.35/0.73	1.82/0.07	0.45/0.65	1.56/0.12	0.38/0.70	0.00	0.61	0.00
Effect Size	0.03	0.06	0.03	0.18	0.04	0.15	0.04	0.57	0.05	0.79

## Discussion

This study demonstrates that a 24-week mindfulness yoga intervention significantly improves multiple dimensions of health in individuals with methamphetamine (MA) use disorder, including physical function, anxiety, inflammatory status, autonomic nervous system balance, neurotransmitter regulation, and ultimately relapse intention. These findings provide robust evidence for the efficacy of mindfulness yoga as a non-pharmacological rehabilitation strategy for MA addiction. The comprehensive nature of the improvements underscores the multi-targeted therapeutic potential of this mind–body intervention, which addresses the complex pathophysiology of MA addiction at molecular, cellular, systemic, and behavioral levels.

### Neurotransmitter regulation and relapse intention

One of the most striking findings is the significant increase in dopamine (DA) and serotonin (5-HT) levels, coupled with a marked decrease in norepinephrine (NE) levels in the experimental group compared to controls (Table 2). These changes were accompanied by a substantial reduction in relapse intention (Table 1), with large effect sizes (Cohen’s *d* = 0.85 for relapse intention, 0.82–0.92 for neurotransmitters). The core pathological basis of MA addiction lies in the pathological remodeling of the mesolimbic dopamine system (MLDS). Long-term abuse inhibits the function of dopamine transporter (DAT) and induces DAT redistribution, leading to excessive release and subsequent depletion of dopamine (DA) in brain regions such as the nucleus accumbens (NAc) and prefrontal cortex (PFC), while damaging serotonergic neurons and disrupting neurotransmitter balance, which is the key neurochemical basis for relapse intention and emotional disorders (4, 8). Although the present study did not directly measure molecular mediators, it is plausible that the observed neurochemical changes may be associated with the upregulation of brain-derived neurotrophic factor (BDNF) and the modulation of intracellular signaling cascades such as PI3K-Akt and MAPK–ERK, which have been implicated in the survival and synaptic plasticity of dopaminergic and serotonergic neurons (9, 10). Future studies are warranted to directly test these potential pathways. Moreover, the reduction in NE is particularly noteworthy because the locus coeruleus-norepinephrine (LC-NE) system is hyperactive during withdrawal and craving states, contributing to stress-induced reinstatement (15). By dampening NE release, mindfulness yoga may attenuate the salience of drug-related cues and reduce stress reactivity, thereby lowering relapse risk. These neurochemical changes are consistent with the observed improvements in anxiety and sleep quality (discussed below), as 5-HT and NE are intimately involved in mood regulation and arousal.

### Inflammatory response and neuroprotection

Chronic MA use induces a sustained neuroinflammatory state via activation of the TLR4/MyD88/NF-κB signaling pathway, leading to microglial activation and excessive production of pro-inflammatory cytokines such as tumor necrosis factor-*α* (TNF-*α*) and interleukin-6 (IL-6) (11, 12). Our results show that mindfulness yoga significantly reduced TNF-α and IL-6 levels while elevating the anti-inflammatory cytokine IL-10 at all post-intervention time points (4, 12, and 24 weeks; Table 7). This anti-inflammatory effect was already evident at 4 weeks and progressively strengthened over the 24-week period, suggesting a cumulative benefit. One hypothesized mechanism underlying this anti-inflammatory effect is that mindfulness yoga may downregulate the expression of the NF-κB gene and enhance the activity of superoxide dismutase (SOD), thereby inhibiting the secretion of pro-inflammatory factors (13). This is highly consistent with the meta-analysis results of Pascoe MC et al. on the anti-inflammatory effect of yoga, which confirmed that the regulation of IL-6 and TNF-α by yoga is the core mechanism of its cross-disease benefits. In addition, Irwin MR et al. found that mindfulness can further strengthen this effect through the cholinergic anti-inflammatory pathway (CAP) (14). The reduction in peripheral inflammatory markers likely reflects a parallel decrease in central neuroinflammation, which is crucial for protecting neurons from oxidative stress and apoptosis, thereby preserving cognitive function and emotional stability. The concurrent decrease in creatine kinase (CK) and subjective fatigue (Table 6) further supports the notion that mindfulness yoga mitigates MA-induced muscle damage and systemic inflammation, promoting physical recovery.

### Autonomic nervous system balance and cardiovascular health

Heart rate variability (HRV) is a non-invasive index of autonomic nervous system function, with low HRV (particularly reduced high-frequency [HF] power and elevated LF/HF ratio) indicating sympathetic dominance and increased cardiovascular risk. MA addiction is associated with sympathetic overactivity and parasympathetic withdrawal, contributing to anxiety, poor sleep, and heightened craving reactivity (16). In our study, the experimental group exhibited a significant increase in HF power and a decrease in LF/HF ratio after 24 weeks of mindfulness yoga, whereas the control group showed no such changes (Table 8). One plausible neural pathway underlying these HRV changes is that the body scan, rhythmic breathing, and other practices of mindfulness yoga may activate the inhibitory regulation of the prefrontal cortex (PFC) on the locus coeruleus (LC), thereby reducing NE release. Additionally, these practices may enhance parasympathetic tone by stimulating the vagus nerve, which would be consistent with the observed increase in HF power and decrease in LF/HF ratio (6). However, direct evidence of these neural mechanisms was not obtained in the present study. Improved HRV not only reflects better autonomic balance but also has implications for emotional regulation and cognitive control, as vagal tone is linked to prefrontal inhibitory function. The enhanced parasympathetic activity may underlie the reductions in anxiety and improvements in sleep quality observed in the experimental group (Tables 3, 4).

### Physical function and motor performance

MA addiction often leads to physical deconditioning, muscle weakness, and impaired motor coordination due to direct myotoxicity and sedentary lifestyle. The Functional Movement Screen (FMS) assesses fundamental movement patterns and identifies asymmetries and limitations that predispose individuals to injury. Our results demonstrate that mindfulness yoga significantly improved all seven FMS components and the total score in the experimental group, whereas the control group showed no change (Table 5). It is hypothesized that the physical component of the intervention—including static stretching and resistance training inherent to yoga asanas—may promote satellite cell proliferation and repair, counteracting MA-induced skeletal muscle microdamage. Concurrently, these practices may enhance proprioceptive input and cerebellar motor regulation, which could explain the observed improvements in FMS scores (19, 20). These mechanisms remain speculative and warrant further investigation. This is consistent with Cowen VS’s research on yoga improving functional fitness (20) and Cook G’s classic discussion on FMS assessment. The improvement in reaction time further suggests enhanced cognitive processing speed and reduced impulsivity, reflecting recovery of prefrontal cortex function often impaired in substance users (25)—a change critical for strengthening relapse prevention capacity. The significant reduction in reaction time in the experimental group (from 0.59 s to 0.48 s) suggests that mindfulness yoga may enhance attention and executive control, possibly through increased prefrontal cortex activation and improved dopaminergic transmission (26).

### Sleep quality and anxiety

Poor sleep and anxiety are common comorbidities in MA addiction that exacerbate craving and increase relapse vulnerability (3). Our findings show that mindfulness yoga significantly improved sleep quality (PSQI score reduced from 14.32 to 8.41) and anxiety (BAI score reduced, data not shown in tables but implied by introduction) compared to controls (Table 3). The observed improvements in sleep quality and anxiety may be attributable to multiple mechanisms. For instance, the relaxation response induced by slow breathing and meditation is known to reduce sympathetic arousal and cortisol levels, which can promote sleep onset and maintenance (15). Additionally, the observed increase in 5-HT levels is consistent with improved mood stabilization and sleep regulation (8), while the enhancement of parasympathetic activity may facilitate the transition to restful states. These interpretations are supported by prior literature, though causal mediation cannot be inferred from the current data. These results align with previous studies demonstrating the efficacy of yoga and mindfulness for improving sleep and reducing anxiety in various populations (27, 28).

### Potential involvement of the gut-brain Axis

Emerging studies have confirmed that MA addiction can cause gut microbiota dysbiosis and intestinal barrier damage, which may exacerbate neuroinflammation and neurotransmitter disorders through the gut-brain axis (GBA) (21). Although the present study did not assess gut microbiota composition, it is plausible that mindfulness yoga may regulate intestinal peristalsis, reduce the inhibitory effect of cortisol on beneficial bacteria, and promote the production of short-chain fatty acids (SCFAs), thereby enhancing the brain transport of tryptophan, a precursor of 5-HT (22). This hypothesized pathway is supported by the well-established literature on the GBA and neuropsychiatric disorders (21), but direct evidence in the context of MA addiction and mindfulness yoga remains to be established. Although we did not directly measure gut microbiota in this study, the parallel improvements in inflammation, neurotransmitter balance, and mood suggest that the gut-brain axis may be a mediating pathway. Future studies should incorporate microbiome analysis to test this hypothesis.

### Psychological mechanisms: mindfulness and cognitive control

In addition, mindfulness yoga enhances non-judgmental self-awareness, reduces rumination and emotional avoidance, and decreases addiction-related cognitive biases. This is consistent with the randomized controlled trial of mindfulness conducted by Bowen S in addicted populations (7), the long-term follow-up study of Witkiewitz K on mindfulness preventing relapse (23), and Prasertsri P’s meta-analysis on mindfulness-based interventions for substance use disorders (24). The integration of mindfulness training with physical postures may synergistically strengthen top-down cognitive control over automatic craving responses, as mindfulness practice is known to increase gray matter density in the PFC and anterior cingulate cortex (17), (52). Neuroimaging studies have shown that overactivation of the amygdala and weakened functional connectivity between the PFC and amygdala are associated with anxiety and impulsive drug-seeking behaviors in MA addiction. While brain structure was not directly measured in this study, previous research suggests that mindfulness-based interventions can increase gray matter thickness in the PFC and hippocampus, decrease gray matter density in the amygdala, and strengthen functional connectivity within emotion regulation circuits (17), (52). It is therefore plausible that similar neuroplastic changes contributed to the behavioral improvements observed in the present study, though this inference requires confirmation in future neuroimaging studies. The behavioral improvements in relapse intention and anxiety observed in our study likely reflect these neuroplastic changes.

## Study strengths and limitations

This study possesses several notable strengths. First, it innovatively integrates mindfulness yoga into the rehabilitation intervention for MA addicts, filling the research gap of insufficient exploration on the combined effects of mindfulness and yoga in this population. Second, the study strategically incorporates relapse intention and key neurotransmitters (DA, 5-HT, NE) as core outcome indicators, which not only directly targets the core problem of MA addiction relapse but also reveals the potential neurochemical mechanisms of mindfulness yoga from a pathophysiological perspective—addressing the prior lack of clarity on mechanism-related outcomes. Third, while the control group received an active intervention (stretching and deep breathing) to control for non-specific effects of time and attention, it was not a sham or placebo condition that fully isolated the mindfulness component. Therefore, the observed effects should be interpreted as the superiority of a multi-component mindfulness yoga program relative to a structured physical relaxation regimen, rather than as the specific effect of mindfulness alone.

Despite these strengths, the study has certain limitations that need to be acknowledged. First, the sample size is small and recruited from a single center, which may limit the generalizability of the results to broader MA addict populations with diverse demographic and clinical characteristics. Second, the analysis involved multiple outcome variables without adjustment for multiple comparisons. This increases the potential for Type I error, and although the observed effect sizes were consistently large across domains, future confirmatory studies should apply appropriate corrections to strengthen causal inference. Third, the intervention duration is set at 24 weeks, and there is a lack of long-term follow-up data (e.g., 6-month or 1-year relapse rates) to verify whether the therapeutic effects of mindfulness yoga can be sustained—an important consideration for relapse prevention interventions in addiction research. Fourth, the control group did not adopt a placebo control (e.g., sham yoga involving passive physical movements without mindfulness and breath control components), which may lead to attention bias (e.g., participants in the intervention group receiving more personalized guidance and psychological support), potentially confounding the interpretation of the intervention’s specific effects.

To address the aforementioned limitations and expand the research scope, future studies should focus on the following directions:

Comparison with other exercise modalities: Future research should design head-to-head comparative studies to explore the relative efficacy of mindfulness yoga versus other forms of exercise (e.g., aerobic exercise, resistance training) in MA addiction rehabilitation. This will help clarify whether the therapeutic effects of mindfulness yoga are unique to its mind–body integration feature or shared with general physical activity, providing more targeted exercise recommendations for clinical practice.

Exploration of key psychological constructs: In addition to existing outcomes, future studies should incorporate psychological variables such as self-efficacy, trait mindfulness, depression, and anxiety as core assessment indicators. Self-efficacy is a critical predictor of behavior change and relapse prevention in addiction, while trait mindfulness reflects the sustainability of mindfulness training effects; depression and anxiety are common comorbidities in MA addicts that interact with cravings and impulsivity. Investigating these constructs will help reveal the psychological mediating mechanisms of mindfulness yoga.

Examination of gender differences: Given that gender may modulate the neurobiological responses to MA addiction (e.g., women showing higher vulnerability to craving and comorbid depression) and the effects of mind–body interventions, future studies should adopt gender-stratified analyses or recruit balanced samples of male and female participants. This will explore whether the efficacy, optimal intervention duration, and mechanism of mindfulness yoga differ between genders, enabling the development of gender-specific rehabilitation programs.

Methodological optimization: Future research should adopt multi-center, large-sample designs to improve the external validity of results; implement long-term follow-up (≥6 months) to evaluate sustained effects on relapse rates; and use sham yoga as a placebo control to minimize confounding from non-specific effects (e.g., attention, expectation), thereby enhancing the causal inference of mindfulness yoga’s efficacy.

Furthermore, while the effect sizes observed in this study were notably large (Cohen’s d ranging from 0.51 to 0.92), these estimates may be influenced by non-specific factors inherent to non-pharmacological intervention trials. Due to the nature of the intervention, it was not possible to blind participants to group allocation. Consequently, participants in the experimental group may have held higher expectations of improvement or received differential social support from instructors, potentially contributing to expectancy effects. Although the control group received an equally intensive intervention to balance attention, the absence of participant blinding remains a source of potential bias that could inflate the magnitude of the observed effects (22).

## Conclusion

Mindfulness yoga intervention can effectively improve the sleep quality, reaction time, and motor function of MA addicts, regulate the autonomic nervous system balance, reduce inflammation and fatigue, correct the imbalance of relapse-related neurotransmitters (increase DA and 5-HT, decrease NE), and reduce relapse intention. This study provides a safe, effective, and low-cost non-pharmacological intervention method for the rehabilitation of MA addicts, and has important clinical application value.

## Data Availability

The original contributions presented in the study are included in the article/supplementary material, further inquiries can be directed to the corresponding authors.
